# Empowering community-based maternal health research: insights from the National Institutes of Health IMPROVE initiative

**DOI:** 10.3389/fpubh.2025.1667629

**Published:** 2025-09-25

**Authors:** Balkissa S. Ouattara, Maurice Davis, Damiya Whitaker, Sung Sug Yoon, Juanita Chinn, Elizabeth Neilson, Elizabeth Barr, Alison Cernich

**Affiliations:** National Institutes of Health, Bethesda, MD, United States

**Keywords:** National Institutes of Health, improve, challenge, maternal health research, community-based organizations

## Abstract

In September 2022, the National Institutes of Health (NIH) launched the Connecting the Community for Maternal Health (CCMH) Challenge as part of the Implementing a Maternal Health and Pregnancy Outcomes Vision for Everyone (IMPROVE) initiative (12). The IMPROVE initiative prioritizes research to reduce preventable maternal mortality, mitigate severe maternal morbidity, and address health disparities. The CCMH challenge sought to reduce maternal health disparities by enhancing research capacity within community-based organizations. Through training, mentorship, research resources, and cash awards, the CCMH challenge empowered local organizations to engage in maternal health research that directly addressed the needs of their communities. By increasing access to NIH resources, the challenge positioned these organizations to contribute sustainably to improved maternal health outcomes. This article provides practical insights into how public health institutions can support community-based research and cultivate partnerships to reduce maternal health disparities. It outlines the competition’s structure, strategies, and outcomes while highlighting key implementation challenges and lessons learned.

## Introduction

Despite progress in obstetric care, maternal health disparities, particularly among underserved communities, remain a critical public health issue in the United States. Black women, American Indian and Alaska Native women, and women in rural areas and from low-income backgrounds are disproportionately affected by adverse health outcomes, including higher rates of maternal mortality, morbidity, and complications during pregnancy and childbirth (1, 2). These disparities are driven by limited access to quality healthcare and other socioeconomic barriers (3, 4). Evidence suggests that the promotion of community-driven solutions that reflect the unique needs of local populations is needed to more effectively address these disparities (5, 6). However, few studies have evaluated the impact of community-based organization (CBO)-led maternal health programs. Many CBOs lack key infrastructure and resources in areas such as research training, data collection and analysis capabilities, access to funding, and expertise in ethical considerations and human subject protection. These barriers limit their ability to conduct rigorous research that can improve health and healthcare practices or inform policy (7). To bridge this gap, the National Institutes of Health (NIH) launched the Connecting the Community for Maternal Health (CCMH) Challenge through its Implementing a Maternal Health and Pregnancy Outcomes Vision for Everyone (IMPROVE) initiative (15). The CCMH challenge was designed to acknowledge the unique expertise of community-based organizations and provide them with the tools and support (training, mentorship, research resources, and cash awards) necessary to engage in impactful maternal health research and to increase competitiveness in pursuit of federal funding opportunities. Importantly, the development of the CCMH challenge modular framework incorporated direct input from CBOs. Community partners were involved throughout the challenge design and implementation to ensure that the structure, training opportunities, and mentorship model aligned with their priorities and addressed barriers they had identified. For instance, the selection of topics for training webinars and workshops was guided by CBOs feedback, ensuring that the content was both relevant and applicable to their organizational contexts. This manuscript outlines the CCMH challenge’s design, implementation, outcomes, and challenges while focusing on its role in building capacity among CBOs to conduct impactful maternal health research.

## Context

The CCMH challenge targeted 501(c)(3) organizations; 501(c) refers to a section of the United States Internal Revenue code that designates specific types of non-profit organizations exempt from federal income tax. Organizations qualifying under 501(c)(3) are typically charitable, religious, educational, and scientific, among others, that operate for public benefit and do not distribute profits to individuals (8). Eligible organizations were not degree-granting educational institutions, prioritizing organizations operating in high-disparity regions, including rural areas, urban underserved communities, and tribal lands in the United States Tribal lands in the United States—such as reservations and trust lands—are federally designated for Native American tribes. They are managed by tribal governments and hold a distinct sovereign status under U.S. law (9–11). This geographical diversity ensured the program addressed maternal health challenges across varied social and structural landscapes. Furthermore, the CCMH challenge engaged populations with maternal health issues, focusing on innovative approaches that integrated mental health. By tailoring these strategies to specific community contexts, the CCMH challenge advanced maternal health outcomes and underscored the importance of community-centered solutions.

## Key programmatic elements

The CCMH challenge followed a three-phase model and spanned 24 months, from September 2022 to September 2024, providing participating CBOs with the time and resources needed to build capacity, implement research projects, and report outcomes effectively.

### Gathering phase: recruitment

The CCMH challenge was launched in September 2022 with a nationwide call for applications from community-based, non-profit, organizations interested in maternal health research. Initially, participants engaged in three virtual workshops during which an overview of the Challenge was provided, and eligibility criteria discussed. Participants had to attend the initial webinar to be eligible to apply for the Challenge. The sessions also included guidance on conducting organizations’ internal assessments and preparing initial applications. More than 90 organizations applied, and 50 were selected to continue participation based on their readiness to engage in maternal health research and existing relationships with the population of focus. These organizations included diverse profiles such as community health centers, grassroots advocacy groups, and social service organizations operating in high-disparity regions.

### Gathering phase: training activity

Subsequently, 45 of the 50 organizations took part in four additional webinars aimed at enhancing their understanding of hypothesis development, research design, and infrastructure to support a research study. Topics cover foundational knowledge such as understanding the NIH, the Challenge design, deconstructing NIH proposals, conducting literature reviews, health problems concept mapping, forming hypotheses, and maximizing research with limited resources. This phase also includes practical exercises like case studies and draft reviews. The organizations also received customized coaching from an experienced research investigator with prior NIH funding. During this phase, one-on-one sessions helped them develop and refine their proposals. Participants valued the education and coaching support, with many reporting that they felt better equipped to develop significant, achievable and competitive research projects in the future. Submissions at the end of the gathering phase were reviewed in several rounds by federal employees serving as judges and external technical advisors. The judging criteria focused on organizational experience, project relevance to maternal health and the IMPROVE initiative, alignment with the Challenge goals, feasibility, and potential to reduce maternal morbidity and mortality (12). Ultimately, 15 organizations progressed to the proposal phase.

### Proposal development phase

The proposal development phase included a series of nine virtual training workshops covering key topics such as specific aims, study design, budgeting, risk management, and timeline development. During the proposal phase, each organization was tasked with developing a full research proposal. Organizations were also introduced to specific aspects of maternal health research, including strategies for recruiting participants and collecting both qualitative and quantitative data. One of the standout features of this phase was the continuity of coaching, with the same coach providing mentorship and transparent guidance to support growth throughout the challenge. This personalized approach helped organizations transform their study design process to meet the rigorous standards required for NIH funding while remaining feasible given the organization’s resources and capacity. Additionally, participants reported in feedback surveys that the interactive coaching helped them navigate challenges such as staff turnover, partnership with academic institutions, and internal resource limitations, which are common issues faced by community-based non-profits. At the end of the proposal phase, 14 organizations submitted complete research proposals (one organization did not meet the submission criteria), which were evaluated according to pre-defined judging criteria (12). Of those, nine organizations were selected to advance to the research phase. The judging criteria for admission to the research phase prioritized research significance, rationale, activities, outcomes, risk mitigation, expertise, and project planning. Proposals were primarily evaluated on the novelty, feasibility, and relevance of the research idea, its clarity and connection to community needs, and the strength of the supporting rationale. Reviewers assess whether the research activities are feasible, aligned with the timeline and budget, and whether anticipated outcomes are consistent with **IMPROVE** objectives around addressing disparities in maternal mortality and morbidity. Proposals also had to identify key risks with appropriate mitigation strategies and demonstrate a clear understanding of necessary resources and plans for acquisition. Finally, a realistic timeline and budget that align with the overall project plan were essential for a strong submission.

### Research phase

This phase was the most intensive and critical part of the CCMH challenge and included the conduct of individual research projects proposed in the previous phase. Thirty-five training webinars over this period emphasize essential research skills like data collection and analysis, manuscript writing, project management, and grant writing, and provides opportunities for learning from symposiums and guest presenters. In addition to that content, hands-on guidance on ethical considerations, and managing institutional review board (IRB) approvals was provided. Throughout the research phase, individualized open-ended coaching sessions were held, allowing organizations to discuss any challenges they encountered, including recruitment difficulties, data collection issues, and project management hurdles. This flexibility ensured that each organization received the support needed to successfully complete their proposed research and strengthen their research infrastructure and capacity. Most of the participating organizations were unfamiliar with research ethics and human subject protection in research. Accordingly, a major feature of this phase was IRB support provided by NIH through an external research services review organization that offered continuous guidance on managing ethical considerations.

At the conclusion of the research phase in June 2024, the eight organizations that remained in the competition submitted their final research reports and showcased their findings during a virtual event.

### Final presentation and evaluation

Eight organizations presented at the CCMH showcase event in July 2024 to summarize their research process, preliminary findings, and potential implications of their work for maternal health in their communities. The presentations demonstrated the breadth of topics explored, ranging from the effects of socioeconomic stressors on maternal outcomes to the role of healthcare access in pregnancy complications. Event attendees included NIH leaders, other maternal health partners, and the public.

The judging process based on the research project reports focused on the scientific rigor of the research, and the overall impact of the findings on maternal health, as well as progress in capacity building (12). All eight organizations reached or exceeded the threshold for prize eligibility that required scoring in the 75th to 100th percentile (13). Table 1 highlights the Research Phase funded projects.

**Table 1:** Research phase funded projects—preliminary outcomes.

Organization	Project title	Preliminary outcomes
Nurturely	Carrying for the Culture: An Infant Carrying Intervention for Health Equity	After taking a culturally informed infant-carrying training, perinatal care providers reported higher levels of infant carrying confidence and knowledge
Central Jersey Family Health Consortium	Can It Happen to Me?	Used a national dataset to develop an algorithm for predicting risk for severe pregnancy-related complications based on pre-pregnancy medical records
The Abundance Project	Impact of Postpartum Doulas on Mental Health and Hypertension in BIPOC Mothers	Receiving postpartum doula care was associated with a reduction of depression and anxiety symptoms
Atlanta Birth Center	Promoting Healing Centered Tools for Pregnancy, Birth, and Beyond	Participation in the Community Resilience Model (CRM) was associated with an improvement in patient-reported resiliency
Buffalo Prenatal Perinatal Network, Inc.	Survivor Moms’ Companion: A Perinatal Post-Traumatic Stress Disorder Program	After participating in the Community Health Workers delivered program, statistically significant reductions in post-traumatic stress disorder- and interpersonal sensitivity symptoms were noted.
Claris Health	Maternal Health Literacy for Nutrition Care Equity	After providing tailored nutrition education and increased access to dietitians in underserved communities, monitoring and support of at-risk pregnancies improved helping to reduce maternal morbidity and mortality.
Doula CO-OP of Nevada	Culturally Congruent Doula Care: The Missing Lens into Maternal Mental Health	After providing culturally congruent doula care matching for labor and delivery services, birthing experience satisfaction, and physical and mental health outcomes improved.
HealthConnect One	Acceptability of Mental Health First Aid Training Among Community Doulas to Reduce Perinatal Mood and Anxiety Disorders and Maternal Mortality in the U.S.	Mental Health First Aid Training increased doulas’ comfort and clarity in addressing mental health issues and their ability in connecting clients with resources.

## Discussion

### Practical implications

The CCMH Challenge sought to create a paradigm shift in how maternal health disparities are addressed. Recognizing the limitations of traditional research paradigms—which often exclude community-based programs—the Challenge aimed to democratize research by directly supporting CBOs. Through a comprehensive program of capacity building, mentorship, and prize competition, the Challenge successfully empowered local organizations to conduct research that was both relevant to their populations and aligned with the scientific priorities of the IMPROVE initiative, which emphasizes interdisciplinary approaches to reducing maternal mortality and morbidity (14).

Quantitative and qualitative metrics were used to evaluate the Challenge’s impact on capacity building, grant competitiveness, and community impact. Eight organizations successfully carried out community-specific research projects, each designed to address unique maternal health needs, demonstrating the Challenge’s effectiveness in building research capacity and infrastructure. In addition, these projects have the potential to create measurable community impacts, such as enhanced postpartum care, improved maternal well-being, and expanded access to services, all of which contribute to reducing disparities in maternal health outcomes. Participants also reported, through feedback surveys, significant skill development in research design and execution—such as formulating testable hypotheses and creating robust data collection protocols. These skills enhanced the methodological rigor of their projects, reflected in more detailed statistical analyses and refined participant recruitment strategies.

As a result of the skills gained in research and grant writing, these organizations saw increased grant competitiveness, with one participating organization securing a first multi-year contract with a local health department to expand its maternal health intervention and another leveraging its experience to submit an NIH grant proposal. These accomplishments highlight the Challenge’s potential to establish a sustainable research funding pipeline. Overall, the Challenge positioned these organizations as leaders in community-centered maternal health research, improving their operational strategies and enabling them to advocate for policy changes informed by their research.

### Lessons learned for future applications

The CCMH Challenge established a strong foundation for sustained community-driven research by equipping organizations with essential tools, fostering collaborative networks, and employing a flexible, replicable program design. Training and mentorship provided CBOs with critical research skills, enhancing their capacity to independently develop and implement future projects while fostering a culture of innovation within underserved communities. Collaborative networks connected CBOs with academic institutions and federal agencies, enabling knowledge exchange and ongoing support beyond the program’s duration.

The program’s adaptable modular framework makes it applicable to other public health areas, such as mental health or chronic disease prevention. However, sustaining these achievements requires continued mentorship and funding, streamlined IRB processes for community-led projects, and improved engagement strategies to address participant recruitment challenges. These efforts are essential to building on the progress of the CCMH Challenge and advancing community-based research in the long term.

### Acknowledgments of constraints

The CCMH challenge, though impactful, faced challenges that underscored the complexities of community-based maternal health research. Delays in obtaining IRB approvals proved a major hurdle, particularly for CBOs new to research ethics, compressing timelines and limiting data collection. Participant recruitment also posed difficulties, with logistical barriers like transportation, childcare, and work schedules hindering engagement in under-resourced communities. Additionally, the 12-month research phase, while valuable, was insufficient for some CBOs to collect comprehensive data or conduct longitudinal analyses, limiting the ability to evaluate long-term outcomes or address complex social determinants of health. These challenges highlighted the need for more streamlined processes, enhanced recruitment strategies, and extended timelines to optimize the impact of such initiatives.

## Data Availability

The original contributions presented in the study are included in the article/supplementary material, further inquiries can be directed to the corresponding author/s.

## References

[1] Howell EA. Reducing disparities in severe maternal morbidity and mortality. Clin Obstet Gynecol. (2018) 61:387–99. doi: 10.1097/GRF.0000000000000349, PMID: 29346121 PMC5915910

[2] Petersen EE Davis NL Goodman D Cox S Mayes N Johnston E . Racial/ethnic disparities in pregnancy-related deaths—United States, 2007–2016. MMWR Morb Mortal Wkly Rep. (2019) 68:762–5. doi: 10.15585/mmwr.mm6835a3 31487273 PMC6730892

[3] Bailey ZD Krieger N Agénor M Graves J Linos N Bassett MT. Structural racism and health inequities in the USA: evidence and interventions. Lancet. (2017) 389:1453–63. doi: 10.1016/S0140-6736(17)30569-X 28402827

[4] Wallace M Crear-Perry J Richardson L Tarver M Theall K. Separate and unequal: structural racism and infant mortality in the US. Health Place. (2022) 73:102736. doi: 10.1016/j.healthplace.2021 28363132

[5] Baciu A Negussie Y Geller A Weinstein JN. Communities in action: Pathways to health equity. Washington, DC: National Academies Press. (2017). 28418632

[6] Williams DR Purdie-Vaughns V. Needed interventions to reduce racial/ethnic disparities in health. J Health Polit Policy Law. (2016) *41*:627–51. doi: 10.1215/03616878-3620857, PMID: 27127267

[7] Centers for Disease Control and Prevention (CDC), Agency for Toxic Substances and Disease Registry (ATSDR) (2011) Principles of community engagement (second edition). Available online at: https://www.atsdr.cdc.gov/community-engagement/php/about/ (Accessed April 20, 2025).

[8] Internal Revenue Service (IRS) (2023) Exemption Requirements - 501(c)(3) Organizations. Available online at: https://www.irs.gov/charities-non-profits/charitable-organizations/exemption-requirements-501c3-organizations (Accessed August 25, 2025).

[9] Congressional Research Service. (2021). Types of Indian land ownership: Trust, restricted fee, and fee simple (CRS in focus no. IF11944). Federation of American Scientists. Available online at: https://sgp.fas.org/crs/misc/IF11944.pdf (Accessed August 25, 2025).

[10] U.S. Department of the Interior. (n.d.). What is a reservation? Bureau of Indian Affairs. Available online at: https://www.bia.gov/frequently-asked-questions (Accessed August 25, 2025).

[11] U.S. Department of the Interior. (n.d.). Tribal sovereignty. Available online at: https://www.doi.gov/ocl/tribal-sovereignty-ruling (Accessed August 25, 2025).

[12] Challenge.gov. (2022). Connecting the Community for Maternal Health Challenge. Available online at: https://www.challenge.gov/?challenge=community-maternal-health (Accessed April 20, 2025).

[13] National Institutes of Child Health and Human Development (NICHD) (2024) Item of interest: NIH announces final winners of the connecting the Community for Maternal Health Challenge. Available online at: https://www.nichd.nih.gov/newsroom/news/090524-winners-connecting-community-maternal-health-challenge (Accessed April 20, 2025).

[14] National Institute of Child Health and Human Development (NICHD) (n.d.) About the IMPROVE initiative. Available online at: https://www.nichd.nih.gov/research/supported/IMPROVE/about (Accessed August 25, 2025).

[15] Bianchi DW Clayton JA Zenk SN. Addressing the public health crisis of maternal mortality: a national research agenda. JAMA. (2023) 330:1729–30. doi: 10.1001/jama.2023.21294, PMID: 37831443 PMC11742278

